# Identifying and understanding how people living with a lower-grade glioma engage in self-management

**DOI:** 10.1007/s11764-023-01425-x

**Published:** 2023-07-14

**Authors:** Ben Rimmer, Michelle Balla, Lizzie Dutton, Joanne Lewis, Morven C. Brown, Richéal Burns, Pamela Gallagher, Sophie Williams, Vera Araújo-Soares, Tracy Finch, Fiona Menger, Linda Sharp, on behalf of the Ways Ahead study team

**Affiliations:** 1 Population Health Sciences Institute, Newcastle University Centre for Cancer, Newcastle University, Newcastle Upon Tyne, England; 2 Faculty of Medical Sciences, Newcastle University, Newcastle Upon Tyne, England; 3 Newcastle Upon Tyne Hospitals NHS Foundation Trust, Newcastle Upon Tyne, England; 4 Faculty of Science, Atlantic Technological University, Sligo, Ireland; 5 Health and Biomedical Strategic Research Centre, Atlantic Technological University, Sligo, Ireland; 6 School of Psychology, Dublin City University, Dublin, Ireland; 7 Centre for Preventive Medicine and Digital Health, Department for Prevention of Cardiovascular and Metabolic Disease, Medical Faculty Mannheim, Heidelberg University, Heidelberg, Germany; 8 Department of Nursing, Midwifery and Health, Northumbria University, Newcastle Upon Tyne, England; 9 School of Education, Communication and Language Sciences, Newcastle University, Newcastle Upon Tyne, England

**Keywords:** Lower-grade glioma, Self-management, Wellbeing, Qualitative

## Abstract

**Purpose:**

Lower-grade gliomas (LGG) are mostly diagnosed in working-aged adults and rarely cured. LGG patients may face chronic impairments (e.g. fatigue, cognitive deficits). Self-management can improve clinical and psychosocial outcomes, yet how LGG patients self-manage the consequences of their tumour and its treatment is not fully understood. This study, therefore, aimed to identify and understand how LGG patients engage in the self-management of their condition.

**Methods:**

A diverse group of 28 LGG patients (age range 22–69 years; male *n* = 16, female *n* = 12; mean time since diagnosis = 8.7 years) who had completed primary treatment, were recruited from across the United Kingdom. Semi-structured interviews were conducted. Informed by a self-management strategy framework developed in cancer, directed content analysis identified and categorised self-management types and strategies used by patients.

**Results:**

Overall, 20 self-management strategy types, comprising 123 self-management strategies were reported; each participant detailed extensive engagement in self-management. The most used strategy types were ‘using support’ (*n* = 28), ‘creating a healthy environment’ (*n* = 28), ‘meaning making’ (*n* = 27), and ‘self-monitoring’ (*n* = 27). The most used strategies were ‘accepting the tumour and its consequences’ (*n* = 26), ‘receiving support from friends (*n* = 24) and family’ (*n* = 24), and ‘reinterpreting negative consequences’ (*n* = 24).

**Conclusions:**

This study provides a comprehensive understanding of the strategies used by LGG patients to self-manage their health and wellbeing, with a diverse, and substantial number of self-management strategies reported.

**Implications for Cancer Survivors:**

The findings will inform the development of a supported self-management intervention for LGG patients, which will be novel for this patient group.

**Supplementary information:**

The online version contains supplementary material available at 10.1007/s11764-023-01425-x.

## Introduction

Lower-grade gliomas (LGG) are a subgroup of brain tumours, most commonly diagnosed in working-aged adults [1]. LGGs are rarely cured and typically recur or progress to a high-grade glioma [2]; life expectancy is limited to approximately 5–15 years, depending on the subtype [1, 3]. LGG patients can experience a diverse, often co-occurring, range of tumour-specific (e.g. cognitive impairment, seizures, personality changes, and mobility issues) and more general cancer-related symptoms (e.g. fatigue, pain) [4]. These, in turn, can contribute to changes in social roles, daily functioning, and loss of independence [5, 6].

There is a large and growing evidence base in cancer to suggest that self-management can improve clinical, psychosocial, and health economic outcomes [7]. Self-management in cancer is defined as ‘awareness and active participation by the person in their recovery, recuperation, and rehabilitation to minimise the consequences of treatment, promote survival, health, and wellbeing’. [8] For successful self-management, individuals require a set of behavioural and emotional regulatory skills (e.g. problem solving, decision making), supported by mechanisms of action driven by motivation and confidence [9].

Yun et al. developed a self-management framework [10] that has been extended to head and neck [11] and childhood cancer survivors [12], which identified and categorised the numerous strategy types (i.e. an individual’s approach to self-management) and strategies (i.e. how an individual implements their approach to self-management) used by people living with and beyond cancer. However, little is known about how LGG patients self-manage their condition. It is important that elements of self-management interventions are designed to meet the specific needs of the target population. Existing interventions have typically been developed for specific cancers (e.g. breast) and, consequently, lack adaptability [13]. For LGG patients, living long-term with the emotional impact of an incurable condition, and tumour-specific impairments (e.g. cognitive deficits), may influence both what, and how, self-management strategies are used.

Only two qualitative studies have explored self-management in LGG patients; both reporting few self-management strategies due to these studies narrow focus on coping and adapting [14, 15]. Affronti et al.’s [14] limited inclusion of patients up to 6-months post-diagnosis is restrictive, as people may need more time to accept and adapt to their condition [16], and the strategies needed to self-manage are likely to change over time. Self-management in the longer-term is likely especially important, as people attempt to return to a perceived ‘normality’, beyond support from their care team (e.g. return to work, regaining independence). Though Edvardsson et al.’s [15] participants were, on average, 16 years since diagnosis, most were grades 1–2; grade 1 tumours are distinct from LGGs and have a more favourable prognosis [2]. Consequently, further research is needed to investigate the strategies used to self-manage living with an LGG.

Online self-management resources for adult brain tumour patients largely encompass the active treatment period [17]; hence, a comprehensive understanding of self-management in LGG patients post-treatment would be beneficial to outline the areas where targeted information and advice is required. This study, therefore, aimed to identify and understand how LGG patients engage in the self-management of their condition, post-treatment.

## Method

### Design

This cross-sectional, qualitative study, part of the wider Ways Ahead project [18] was reviewed and approved by the Wales Research Ethics Committee (REC ref: 20/WA/0118). The present analysis focused on identifying and understanding the self-management strategies used by LGG patients.

### Participants and recruitment

Participants were adult LGG patients who lived in the United Kingdom (UK). Individuals were eligible if they were aged ≥ 18 years at diagnosis and were in remission following completion of primary treatment, or stable under observation, following an LGG diagnosis, specifically a grade 2 astrocytoma, or a grade 2 or 3 oligodendroglioma [19]. Participants were ineligible if they were non-English speaking or perceived by a health professional at collaborating National Health Service (NHS) sites to have severe psychological or social problems where participation could risk causing further distress.

Potentially eligible patients were identified through collaborating NHS sites and networks of the Brain Tumour Charity, a leading brain tumour charity based in the UK. Purposive sampling ensured a range of age, sex, diagnoses, and time since diagnosis (< 5, 5–10, > 10 years). At NHS sites, patients were identified from their medical records and provided with a study information sheet by a health professional during a clinic visit. For recruitment via the Brain Tumour Charity, a study flyer and participant information sheet was circulated through newsletters. People were asked to call or email the study team to register their interest. BR and LD called interested patients to confirm eligibility, afford the opportunity for questions, and if the individual was eligible and willing to take part, arrange a convenient interview date and time. Recruitment was conducted between August 2020 and May 2022.

### Data collection

Interviews were conducted by BR and LD, who are trained and experienced in qualitative research. All interviews were conducted remotely using video-conferencing software (e.g. Zoom or Microsoft Teams) or telephone, as per participant preference. Cognitive or communication impairments might impact a patient’s ability to retain or understand questions or provide responses. To facilitate this, we provided a topic overview prior to the interview and allowed ample time for the participant to consider and respond to each question.

Immediately prior to each interview, audio-recorded consent was obtained, and patient-related (including sex, age, employment and relationship status, years of education, number of dependents) and clinical and tumour-related (including diagnosis, date of diagnosis, tumour location and laterality, treatment, IDH1-mutation and 1p19q codeletion status) information was collected. For participants recruited through the Brain Tumour Charity, we asked for their main treating hospital and managing clinician. For all participants, we contacted the treating hospital to confirm the clinical and tumour-related details; where confirmation could not be obtained, the patient-reported information is reported.

Interviews were semi-structured following a topic guide (Online resource 1), which was developed with input from a patient and public involvement panel of brain tumour patients, and clinical colleagues with experience in managing LGG patients (JL & SW). Throughout data collection, any new issues raised were added to the guide, to be explored in subsequent interviews.

Participants were first asked to broadly reflect on life following their LGG diagnosis. How they were impacted by the tumour and its treatment (e.g. cognitive, physical, psychological) and the social and role implications of this impact (e.g. work, transport, relationships, and finances) were then explored. For each area, the interviewer asked probing questions around how the participant managed this impact, and what, and when, support was received or needed. Throughout, participants could raise any additional issues of importance to them.

To thank participants for their time, £20 vouchers were offered. They were also provided with a post-interview sheet with details of charities and helplines, should they have questions at a later date or experience distress. Interviews were audio-recorded and lasted 102 min on average (range 54 to 167 min).

### Data analysis

Interviews were transcribed verbatim and anonymised by an external transcription service, with each participant allocated a unique ID. For accuracy, transcripts were checked against the audio-recordings. Coding and analysis primarily used a deductive, framework-driven approach in line with directed content analysis [20].

The initial categorisation matrix was informed by Brown et al.’s extension of the self-management framework [12], as this was the most recent update and had the most extensive number of strategies. This included 20 self-management strategy types (i.e. an individual’s approach to self-management, such as ‘self-monitoring’), encompassing 133 self-management strategies (i.e. how an individual implements their approach to self-management, such as ‘monitoring emotions’). Concurrent inductive content analysis enabled the identification of new self-management strategies used by LGG patients that were not included in the initial categorisation matrix. Data saturation was determined by the perception that there was sufficient data to support and expand upon the analysis framework [21].

Two trained qualitative researchers (MiB and BR) independently read and coded a random sample of the same six transcripts to the framework. Text that did not map to an existing category was coded as a new category and labelled appropriately. Similarities, differences, and new strategies were discussed, with reference to concrete examples to help distinguish between categories. Remaining transcripts were analysed by MiB, who discussed findings and uncertainties with BR as analysis progressed. The frequency of each strategy type and individual strategy across the interview set is reported in Table 2; illustrative quotes are provided in Online resource 2 to outline LGG patients’ engagement in self-management. To provide greater understanding and illustration of how LGG patients are distinct in their use of self-management, we also report how participants described using and experiencing the most common self-management strategy types.

## Results

### Participant characteristics

Thirty-nine LGG patients registered an interest in taking part. Of these, 35 were eligible and 28 were subsequently interviewed (10 recruited through NHS sites and 18 through the Brain Tumour Charity). Reasons for exclusion included: non-completion of primary treatment (*n* = 2); ineligible diagnosis (*n* = 1); and not a UK resident (*n* = 1). The mean age at interview was 50.4 years (range 22–69 years), and 16 participants were male (Table 1). Diagnoses were grade 2 oligodendroglioma (*n* = 10: IDH1-mutant, yes *n* = 7, no *n* = 2, unknown *n* = 1; 1p/19q codeletion, yes *n* = 9, not known *n* = 1), grade 3 oligodendroglioma (*n* = 9: IDH1-mutant, yes *n* = 6, no *n* = 1, unknown *n* = 2; 1p/19q codeletion, yes *n* = 7, not known *n* = 2), and grade 2 astrocytoma (*n* = 9: IDH1-mutant, yes *n* = 6, no *n* = 1, unknown *n* = 2; 1p/19q codeletion, no *n* = 7, not known *n* = 2). Participants had a mean time since diagnosis of 8.7 years (range 1–18 years).

**Table 1:** Lower-grade glioma sample characteristics at time of interview

Characteristic	*n*	Characteristic	Mean (range)
*Diagnosis*^*a*^		*Time since diagnosis (years)*^*a*^	8.7 (1–18)
Grade 2 oligodendroglioma	10	*Full-time education (years)*	15.8 (11–20)
Grade 3 oligodendroglioma	9	*Sex*	***n***
Grade 2 astrocytoma	9	Female	12
*IDH1-mutation status*^*a*^		Male	16
Yes	19	*Age*	
No	4	≤ 40	4
Unknown	5	41–50	8
*1p/19q codeletion status*^*a,b*^		51–60	11
Yes	16	> 60	5
No	7	*Dependents*	
Unknown	5	None	18
*Treatment*^*a*^		One	3
Surgery	28	Two	6
Radiotherapy	22	Three	1
Chemotherapy	17	*Employment status*	
*Tumour location*^*a*^		Full-time employee	8
Frontal	18	Part-time employee	4
Temporal	3	Retired	4
Parietal	3	Medically retired	6
Overlapping regions	3	Unable to work	6
Unknown	1	*Relationship status*	
*Tumour laterality*^*a*^		Married	21
Right hemisphere	13	In a relationship	3
Left hemisphere	15	Single	2
Dominant hemisphere	13	Widowed	2
Non-dominant hemisphere	15		

^a^Clinical and tumour-related details were patient-reported for eight participants

^b^All participants with 1p/19q codeletion were oligodendroglioma patients; all participants without 1p/19q codeletion were astrocytoma patients

### Engagement in self-management

We found evidence for all 20 self-management strategy types in the initial categorisation matrix and did not identify any new strategy types. In total, 123 different self-management strategies were reported by participants. To manage, protect, and improve their health and wellbeing, each LGG patient reported using a wide range of self-management strategies (median 39; range 19–54) within multiple self-management strategy types (median 16; range 9–18) (Table 2; Online resource 2).

**Table 2:** Self-management strategy types and individual strategies used by LGG patients

Self-management strategy types	Self-management strategies^a^	*n*
Acceptance		**26**
Accepting functional, lifestyle, and social changes following the tumour and its treatment	Accepting new health behaviours	6
	Accepting support^+^	3
	Accepting the tumour and its consequences	26
Activity-based coping		**19**
Use or uptake of hobbies or activities to manage one’s wellbeing	Pursuing an existing hobby/activity	19
	Taking up a new hobby/activity	8
Adopting a healthy lifestyle		**26**
Adopting generic health behaviours to boost one’s general physical and/or emotional wellbeing	Adopting a healthy diet	4
	Being physically active in everyday life	19
	Drinking more water	1
	Exercising	16
	Meditating	4
	Sleeping well	1
	Taking medication	18
	Taking vitamins and minerals	1
Behavioural avoidance		**8**
Behavioural strategies which minimise one’s contact with threats to one’s physical and/or emotional wellbeing	Avoiding activities that may cause harm	4
	Avoiding situations that may cause harm	2
	Avoiding uncomfortable social encounters	5
Cognitive avoidance		**20**
Strategies involving the avoidance of thoughts concerning the negative consequences of the tumour and its treatment	Avoiding finding out too much	9
	Avoiding thoughts about the tumour and its consequences	18
	Distracting oneself by keeping busy	2
Conserving emotional energy		**13**
Strategies which enable one to conserve emotional energy in order to better self-manage one’s condition	Having time to yourself	3
	Letting emotions out	4
	Minimising stress	5
	Switching off	1
	Using sleep	5
Conserving physical energy		**18**
Strategies which enable one to conserve physical energy in order to better self-manage one’s condition	Reducing activities	3
	Reducing workload	14
	Taking a break	13
Creating a healthy environment		**28**
Attempts to create an environment which enables effective self-management	Acquiring knowledge about the tumour, treatment and late-effects and available support	19
	Attending follow-up and screening appointments	21
	Collecting materials to aid self-management	9
	Ensuring reliability of health information on the internet	6
	Learning self-management skills	4
	Obtaining resources to aid self-management	16
	Relationship-building with health practitioner	3
	Using external aids to overcome cognitive difficulties^+^	16
	Utilising skills for independent living	3
	Valuing and respecting relationship with care team	3
Goal and action setting		**25**
Use of planning or goal-setting self-management strategies	Coping planning	3
	Planning daily activities	8
	Priority-based planning	7
	Setting future goals	18
	Setting up facilitating conditions	18
Managing others		**22**
Active attempts to effectively manage one’s social relationships following treatment	Being assertive in social encounters	1
	Being open with others about the tumour and its consequences	18
	Keeping others happy	5
	Protecting others from harm	18
Meaning-making		**27**
Interpreting the tumour and its consequences in the broader context of life as a whole	Appreciating health more	4
	Appreciating life more	10
	Appreciating support	15
	Appreciating the importance of family	5
	Appreciating the severity of one’s illness history	7
	Changing one’s image	1
	Finding meaning in work	4
	Giving back	10
	Taking every day as it comes	12
	Wanting to give something back	12
Positive appraisal		**26**
Focusing on positive aspects of one’s immediate situation	Benefit finding	13
	Downward comparison	18
	Reinterpreting negative consequences	24
Proactive problem solving		**18**
Active attempts to solve problems in-the-moment arising from the consequences of the tumour and its treatment	Acting to prevent further complications	7
	Adaptive approaches to ongoing physical consequences of the tumour and its treatment	17
Reasoned decision-making		**23**
Objective decision-making strategies relating to survivor self-management	Considering the benefits of positive health behaviours	9
	Considering pros and cons of self-management	9
	Evaluating effectiveness of self-management	10
	Thinking objectively about negative health behaviours	1
	Thinking objectively about negative thoughts and emotions	9
Seeking normality		**23**
Active attempts to return to normal living following treatment	Carrying out tasks to the best of one’s ability	5
	Choosing when and to whom to disclose illness history	2
	Focusing on doing normal activities	8
	Focusing on getting back to work	13
	Gaining independence	4
	Maintaining independence	3
	Regaining strength	6
	Returning to normal	10
	Testing oneself	1
Self-monitoring		**27**
Active self-monitoring of one’s health, wellbeing and ongoing care	Knowing your body	7
	Monitoring emotions	23
	Monitoring for symptoms of the tumour and late effects	15
	Monitoring general health	4
	Monitoring health behaviours	1
	Monitoring relationship with health professionals	1
	Recognising one’s own limits	21
Self-motivating		**25**
Strategies which help to motivate oneself to effectively self-manage	Being healthy for sake of one’s family	2
	Challenging yourself	2
	Developing confidence and self-efficacy	1
	Drawing on spiritual resources	1
	Drawing strength from past experiences	2
	Employing a determined attitude	14
	Encouraging oneself	6
	Focusing on milestones of survivorship	3
	Interacting with others	1
	Maintaining a positive outlook	20
	Not dwelling on the past	2
	Persevering with healthy behaviours	3
	Recognising the need for motivation and discipline	1
	Taking responsibility for own health	11
	Wanting to stay in good health	1
Self-sustaining		**12**
Strategies which enable one to consistently implement self-management strategies in one’s daily life	Customising dietary practises	2
	Following health practitioner’s advice	4
	Incorporating self-management behaviours into daily routine	8
	Keeping busy to avoid negative behaviours	2
	Maintaining medical equipment	1
Using sense of humour		**6**
Use of humour to manage emotions associated with the negative consequences of the tumour and its treatment	Finding humour in others’ reactions	1
	Laughing about the tumour and its consequences	5
	Using humour to hide insecurities	1
Using support		**28**
Use of appropriate supports to assist in one’s recovery and recuperation following treatment	Companionship from pet	2
	Drawing support from similar other	15
	Giving advice to similar others^+^	7
	Having family and friends to talk to†	7
	Having health professionals to talk to†	16
	Receiving formal support	20
	Receiving support from charities and organisations	19
	Receiving support from care team	17
	Receiving support from family	24
	Receiving support from friends	24
	Receiving support from partner	20
	Receiving support from the workplace	21
	Seeking formal help	17
	Seeking support from care team	13
	Seeking support from charities and organisations^+^	14
	Seeking support from family	1
	Seeking support from friends	2
	Seeking support from the workplace^+^	1

^+^New strategy identified in interviews with LGG patients

^†^Original strategy has been sub-divided into new categories: ‘having family and friends to talk to’ and ‘having health professionals to talk to’ adapted from ‘having someone to talk to’

^a^Strategies in the framework not identified in interviews with LGG patients: accepting social difficulties, avoiding contact with others for possible infection, avoiding negative health behaviours, avoidance of negative relationships, balancing life with health needs, becoming more altruistic, caring less about what others think, dealing with (in)fertility at the right time, ensuring personal hygiene, receiving support from educational provider, reducing negative health behaviours, rewarding oneself, seeking support from partner, treating illness as a project, trying to fit in, wanting to look good

Bold values indicate the number of people that reported at least one of the individual self-management strategies within each strategy type

### Framework revisions

LGG patients reported 117 of the 133 self-management strategies detailed in the initial categorisation matrix [11, 12], meaning evidence for 16 strategies was not found (e.g. *avoiding negative health behaviours*). Labels for self-management strategy types were left intact to facilitate future use of the framework. For several of the interviewees, the term ‘cancer’ did not resonate with them; therefore, labels for 12 strategies were altered to remove or replace ‘cancer’ with ‘the tumour’ or ‘illness’ (e.g. *seeking support from cancer care team* became *seeking support from care team*; *accepting cancer and its consequences* became *accepting the tumour and its consequences*; and *appreciating the severity of one’s cancer history* became *appreciating the severity of one’s illness history*, respectively). Due to the depth of data reported, we separated ‘having someone to talk to’ into two individual strategies, ‘having family and friends to talk to’ and ‘having health professionals to talk to’.

#### New self-management strategies reported by LGG patients

Five novel self-management strategies were identified, within three of the strategy types (meaning the updated framework now includes 139 strategies). Within ‘creating a healthy environment’, 16 participants reported ‘using external aids to overcome cognitive difficulties’ (e.g. using a calendar to remember health appointments). Within ‘acceptance’, three participants reported ‘accepting support’ (i.e. accepting help with something they could manage previously). Three new strategies were identified within ‘using support’, namely ‘giving advice to similar others’ (i.e. sharing tips from their experiences with other patients), ‘seeking support from charities and organisations’, both reported by several participants, and ‘seeking support from the workplace’, reported by one participant.

### Most common self-management strategy types and strategies

Four self-management strategy types were reported by all (or all bar one) participants: ‘using support’ (*n* = 28), ‘creating a healthy environment’ (*n* = 28), ‘meaning making’ (*n* = 27), and ‘self-monitoring’ (*n* = 27); we expand on how LGG patients implemented these strategy types in detail below. A further five strategy types were reported by the majority of participants: ‘acceptance’, ‘adopting a healthy lifestyle’, ‘positive appraisal’ (*n* = 26 each), ‘goal and action setting’, and ‘self-motivating’ (*n* = 25 each).

The most frequently reported self-management strategies were ‘accepting the tumour and its consequences’ (*n* = 26; within strategy type ‘acceptance’), ‘receiving support from friends’ (*n* = 24; within ‘using support’), ‘receiving support from family’ (*n* = 24; within ‘using support’), ‘reinterpreting negative consequences’ (*n* = 24; within ‘positive appraisal’), and ‘monitoring emotions’ (*n* = 23; within ‘self-monitoring’).

#### Strategy type: using support

Many participants reported seeking support from formal sources/networks (e.g. care team, charities, and organisations) to acquire information and assistance with managing the consequences of their illness; only a few reported seeking support from informal networks like family and friends. Several participants noted the benefit of having family/friends or health professionals available to talk to, should they need their support.

I made an appointment speaking to [the Consultant] about all aspects of [the diagnosis] and what would happen maybe further down the line, so I’m a little bit more informed. (Pa37, aged 54, Male, grade 2 astrocytoma, 3 years since diagnosis).

Most participants reported receiving formal support; for example, physiotherapy or counselling to help manage the physical or psychological impact, respectively. Charities (e.g. the Brain Tumour Charity, Maggie’s, Macmillan Cancer Support), were frequently cited as sources of self-management support via their helplines, websites, and centres.

A lot of information I get from the website of the Brain Tumour Charity itself…We’ve been to a couple of the workshop sessions that they held in the early days. There is a supportive community out there. (Pa15, aged 55, Male, grade 2 astrocytoma, 7 years since diagnosis).

The majority of participants described receiving support from friends, family, partners, and their workplace; for example, practical (e.g. housework, transport) and emotional support, with close contacts often adopting caring roles. Partners and spouses played a major role in providing support.

Like I say, you still get lifts to work and stuff like that and driving, yes, and my wife did a lot of it just to keep basically the pressure off [me]. (Pa31, aged 53, Male, grade 2 oligodendroglioma, 14 years since diagnosis).

Several participants reported drawing support from similar others through support groups and forums, alleviating feelings of isolation and promoting camaraderie. This provided some with a welcome opportunity to give advice and share acquired knowledge of available resources as well as to receive support.

We all had our feelings for each other, that we all had the illness in the same place. There was all the people who had helped, or were still helping…their family. That was really nice…I appreciated it. (Pa19, aged 55, Male, grade 3 oligodendroglioma, 5 years since diagnosis).

#### Strategy type: creating a healthy environment

Most participants reported attending follow-up appointments for routine scans and results to maintain contact with their care team and monitor their condition. Many participants detailed acquiring knowledge about the tumour, treatment, available support, and ways to manage health and wellbeing, by accessing reputable charity websites, webinars, and scientific journals. Participants were mindful of ensuring online information was reliable; where questions were raised, a few participants sought clarity from their care team.

That’s a good thing I learnt from that [fatigue] webinar [run by Brainstrust], when you have energy, spend it on things that you want to do… I think I spend far too much energy on doing things that I don’t, necessarily, want to do. (Pa9, aged 22, Male, grade 2 astrocytoma 1 year since diagnosis).

Most participants actively obtained resources to aid self-management; for example, bus passes and rail cards to manage transport while their driving licence was revoked due to the illness. Several participants reported collecting materials, such as books or charity information packs from their hospital to, for example, improve understanding of symptom management, or find out how to arrange financial support.

There’s actually a book I know, written for people with the same low-grade glioma as mine, which has been quite helpful as it covers a lot of similarities with how I feel. (Pa3, aged 45, Male, grade 2 oligodendroglioma, 18 years since diagnosis).

Many participants detailed using external aids to facilitate memory; for example, using dosette boxes, or setting phone reminders and alarms, to manage medication or remember social engagements. A few participants recounted learning new physical and cognitive skills to maintain hobbies and interests.

I’ve got a big calendar that I write everything on in the kitchen. I just keep on top of things that way. (Pa29, aged 51, Female, grade 3 oligodendroglioma, 9 years since diagnosis).

#### Strategy type: meaning-making

Most participants acknowledged the importance of, and appreciation towards, the availability of a support network; some specifically credited family, feeling lucky to have their support.

My wife’s very supportive, she’s very good at seeing when I’m tired and saying. “Go and sit down.” So, that’s how I manage it…it’s been very good to have that support. (Pa5, aged 56, Male, grade 2 oligodendroglioma, 2 years since diagnosis).

Many participants reported a desire to give something back; for example, through research participation or emotional support to newly diagnosed patients. Several had already attempted to give back, describing charity fundraising efforts and volunteering at hospitals and the resulting benefits of this.

I like to help people, if I can share their experience and bring my positive attitude into their life a little bit, then that’s good. (Pa16, aged 69, Male, grade 3 oligodendroglioma, 2 years since diagnosis).

Several participants detailed taking every day as it comes, approaching challenges of their illness gradually, without becoming overwhelmed. To affirm this, some acknowledged the severity of their illness history, expressing a new appreciation for life, their health, and the positive progress they have made.

You’ve got to be thankful…I look back and see where I was and where I am now. (Pa22, aged 43, Female, grade 2 astrocytoma, 16 years since diagnosis).

#### Strategy type: self-monitoring

Most participants reported self-monitoring their health and emotions to identify any issues with, and in some cases, feel control over, their health and wellbeing. Many described strong emotions in relation to their condition, including fear of tumour progression and anxiety, and/or emotional consequences such as being short-tempered; some participants implemented strategies (e.g. taking a break) to control their emotions.

I can feel myself snapping, not being aggressive or anything but just being a bit snappy. I’ll be like, “You’re right”. I need to lie down. (Pa33, aged 45, Male, grade 2 oligodendroglioma, 9 years since diagnosis).

Several participants reported active monitoring for symptoms: for example, being aware of, and acknowledging, how it feels when they are fatigued, or about to have a seizure. Many detailed an awareness of environments or situations that may exacerbate the risk of a seizure; this awareness allowed them to plan accordingly. For some, this extended to monitoring of general health and taking remedial actions.

My speech was slurred. I sat down and I thought, breathe deeply, keep calm, let’s see what happens, this might resolve itself…The discomfort or the lack of control in the jaw and the tingling in the hand, that subsided. (Pa14, aged 66, Male, grade 2 oligodendroglioma, 4 years since diagnosis).

Through self-monitoring, most participants acknowledged that, largely due to fatigue or the chance of having a seizure, they could not manage the same activity levels as before diagnosis.

There are always going to be times when you can’t do things as fully or as well as you would like to because of your condition and it’s just recognising that fact. (Pa36, aged 42, Female, grade 2 astrocytoma, 8 years since diagnosis).

Recognising these limits impacted participants’ perceived capacity to work and maintain hobbies (e.g. gardening). Consequently, many participants reported only engaging in activities they felt were manageable; these fatigue management approaches link with the strategy type ‘conserving physical energy’. Some suggested this was assisted by the feeling that they knew their body.

## Discussion

LGG patients often live long-term with an incurable condition and its wide-ranging consequences; however, little is known about how they manage their health and wellbeing. Understanding whether, and which, self-management strategies are used is important to inform the need for, and development of, self-management interventions. This study aimed to comprehensively identify and understand the self-management strategies used by LGG patients to manage the consequences of their tumour and its treatment.

To classify and categorise reported self-management strategies, we built upon an established self-management framework for cancer survivors [11, 12]. Through this approach, we recognised 20 strategy types and 123 individual strategies used by LGG patients; this encompassed psychological, social, and behavioural approaches to self-management, comprehensively expanding on existing evidence in LGG patients [14, 15]. We found a similar frequency of ‘self-motivating’ and ‘meaning making’ strategy types in LGG patients, compared to head and neck cancer survivors [11], and ‘creating a healthy environment’ and ‘adopting a healthy lifestyle’, compared to young adult survivors of childhood cancer [12]. ‘Using support’ was one of the most common strategy types used by all three patient groups. However, a ‘healthy environment’ for childhood cancer survivors meant acquiring resources to facilitate physical activity (i.e. gym membership), whereas LGG patients favoured resources to facilitate memory and manage fatigue. This underlines the importance of understanding how different strategies are used by different patient populations, as this likely varies with the consequences of each condition (and, perhaps, where the patient group are in their life course).

Encouraging and facilitating self-management in LGG patients is crucial to ensure they can effectively engage in managing their health and wellbeing. People living with a brain tumour may underestimate cognitive, emotional, psychological, and social changes [22]. This could have implications for patient self-management; for example, ‘monitoring emotions’ and ‘recognising one’s own limits’ were amongst the most commonly reported self-management strategies, but patients would need to be aware of their limitations and changes in emotion to effectively engage with these strategies.

It has been noted previously that self-management should not be a solitary activity but rather one that an individual is supported to engage with [23]. The frequency with which patients in this study spoke about support received from (in)formal support networks underlines the influential role of health professionals, family, and friends in self-management [23, 24]. The practical (e.g. help with housework), emotional (e.g. having someone to talk to), and information (e.g. symptom management leaflet) support provided could facilitate the implementation of numerous self-management strategies, such as ‘reducing workload’, ‘letting emotions out’, and ‘setting up facilitating conditions’, respectively. Still, to expedite the benefits of supported self-management, we need to understand whether there are reasons why LGG patients may not seek help from their support networks [25].

Despite the limited life expectancy following an LGG diagnosis, there was a common refrain of optimism, appreciation, and planning for the future amongst many of the most frequently reported self-management strategy types, such as ‘acceptance’ (particularly ‘accepting the tumour and its consequences’), ‘goal and action setting’, ‘meaning making’, ‘positive appraisal’ (particularly ‘reinterpreting negative consequences’), and ‘self-motivating’. These approaches appear to form a substantial part of self-management in LGG patients; if maintained, acceptance in particular, has been associated with reduced levels of distress [26]. Further, consistent with past work on other cancers [27], the data here indicates that LGG patients may experience post-traumatic growth (PTG), as several frequently reported strategies resembled PTG dimensions (e.g. ‘taking every day as it comes’ resonates with ‘appreciation of life’; ‘reinterpreting negative consequences’ resonates with ‘personal strength’).

While engagement in self-management was consistently high, the importance of, and need for, self-management varied for each participant, with a range of strategies used. We do not know whether participants used these strategies before diagnosis, or if they were approaches that they initiated post-diagnosis or had been taught in rehabilitation. We focused on the most common strategy types as people largely reported, and talked about, what has been helpful to them; yet it is important to acknowledge that less common strategy types, such as ‘activity-based coping’ and ‘conserving physical energy’ were sometimes highly valued by those who reported them. It is important to note that, as others have reported, which strategies are most important for an individual may be influenced by wide-ranging clinical (e.g. tumour type) [28], environmental (e.g. strength of support network), or personal (e.g. employment ambitions) factors [29].

Seizure burden is consistently associated with worse quality-of-life in people living with an LGG [4]. It was interesting, therefore, that seizures were not often mentioned in a self-management context; only a few participants spoke about self-monitoring in relation to minimising the chance of having a seizure. It is possible that this is a result of our participants being, on average, 8.7 years from diagnosis; by this time, seizures may have stabilised for many and be well managed with antiepileptic drugs. Indeed, in a study reporting long-term follow-up of LGG patients, mean symptom scores for seizures were low [30]; and 43% of participants reported a decrease in seizure activity from 6 to 12 years since diagnosis [31].

### Implications

Our findings demonstrate that LGG patients use wide-ranging strategies in the long-term self-management of their condition; thus this patient group may be open to interventions to support them to self-manage. Our data highlights the value of self-monitoring and using support, which may aid the general transition to living with a brain tumour [32]. We also identified the importance of strategies used to manage tumour-specific impairments, such as cognitive function and personality changes. Future intervention development might consider education, information, and signposting, supplemented with appropriate behavioural change techniques, to ensure and maintain awareness of what support is available, what can be done to self-manage different symptoms and impairments, and how this might be achieved. An example would be an interactive session with different strategies to self-manage medication adherence, including making a medication intake plan with anchoring to other activities, setting reminders, and suggestions of using tools like dosette boxes in order to, in time, establish a habit.

### Strengths and limitations

Our study benefitted from recruitment across the UK, covering several regions where available support and resources may differ; the availability of formal support may influence the importance of, need for, and ability to implement, different self-management strategies. Semi-structured interviews, and the wide range of topics covered, gave participants the freedom to report their self-management across various contexts, thus capturing what was important to each individual. Since interviews were conducted remotely, participants may have perceived greater anonymity, thus feeling more comfortable and encouraging more disclosure [33].

Efforts were made to facilitate the inclusion of patients with cognitive, speech, language, and communication impairments, following expert advice from a Speech and Language Therapist. However, the demands of an interview may have prevented patients with fatigue or poor cognitive function from registering an interest, or being approached by health professionals. Consequently, the self-management strategies of LGG patients with these symptoms or impairments may be underrepresented. Ways to facilitate their participation (e.g. multiple, shorter interviews to mitigate fatigue, strategies to support communication) should be considered in future research [34]. Due to COVID-19, the need for partial recruitment through the Brain Tumour Charity means we cannot discount the possibility that participants were somewhat self-selected, and were people who more actively engaged in self-management, had higher levels of self-efficacy, and/or particularly valued support groups/networks. Although we did not formally seek to compare participants recruited through different routes; post-hoc analysis suggested there is little difference in the strategy types or experiences reported. Despite repeated attempts to contact the clinical care teams of participants recruited through the Brain Tumour Charity for confirmation of clinical and tumour-related details, this information was patient-reported for eight participants and IDH1 mutation and 1p19q codeletion status were unknown for five participants.

## Conclusions

This study provides, for the first time, a comprehensive understanding of the strategies used by LGG patients to self-manage their health and wellbeing, post-treatment. LGG patients reported using an extensive and diverse range of self-management strategies, indicating a willingness to engage in self-management. The most common approaches to self-management included the use of support from (in)formal networks, and creating an environment that facilitates effective self-management. How LGG patients implemented their approach to self-management was distinct from other cancer survivors, favouring strategies that facilitated tumour-specific symptoms and impairments (e.g. memory deficits). These findings are valuable to inform the development of supported self-management interventions for this largely neglected patient group [35].

## Supplementary information

Below is the link to the electronic supplementary material.

Supplementary file1 (DOCX 46 KB)

Supplementary file2 (DOCX 100 KB)

## Data Availability

The data that support the findings of this study may be available from the Chief Investigator (Professor Linda Sharp; linda.sharp@ncl.ac.uk) upon reasonable request.
