# Supplementary material for: Identifying and understanding how people living with a lower-grade glioma engage in self-management
Source: J Cancer Surviv. 2023 Jul 14;18(6):1837–50. doi: 10.1007/s11764-023-01425-x (PMC11502583; doi:10.1007/s11764-023-01425-x)
Supplement: Supplementary file 2 — Supplementary file2 (DOCX 100 KB) [file 11764_2023_1425_MOESM2_ESM.docx]

**Identifying and understanding how people living with a lower-grade glioma engage in self-management**

Ben Rimmer^1*^, Michelle Balla^2^, Lizzie Dutton^1^, Joanne Lewis^3^, Morven Brown^1^, Richéal Burns^4,5^, Pamela Gallagher^6^, Sophie Williams^3^, Vera Araujo-Soares^1,7^, Tracy Finch^8^, Fiona Menger^9^, Linda Sharp^1^, on behalf of the Ways Ahead study team^+^

Author affiliations:

1. *Population Health Sciences Institute, Newcastle University, Newcastle University Centre for Cancer, Newcastle, England*
2. *Faculty of Medical Sciences, Newcastle University, Newcastle, England*
3. *Newcastle upon Tyne Hospitals NHS Foundation Trust, Newcastle, England*
4. *Faculty of Science, Atlantic Technological University, Sligo, Ireland*
5. *Health and Biomedical Strategic Research Centre, Atlantic Technological University, Ireland*
6. *School of Psychology, Dublin City University, Dublin, Ireland*
7. *Centre for Preventive Medicine and Digital Health, Department for Prevention of Cardiovascular and Metabolic Disease, Medical Faculty Mannheim, Heidelberg University, Germany*
8. *Department of Nursing, Midwifery and Health, Northumbria University, Newcastle, England*
9. *School of Education, Communication and Language Sciences, Newcastle University, Newcastle, England*

*^+^*The Ways Ahead study team comprises, in addition to the named authors, Sara Erridge, Pauline Sturdy, and Catherine McBain.

**Corresponding author: Ben Rimmer, Population Health Sciences Institute, Newcastle University, Ridley Building 1, Newcastle upon Tyne, NE1 7RU.
Email: ben.rimmer@newcastle.ac.uk; Phone: 0044 (0)7704 300 509*

Online resource 2. Content codes, definitions and examples for self-management strategy types and strategies

| Strategy types with definition | Specific strategies | N | % | Illustrative quotes |
| --- | --- | --- | --- | --- |
| 1. Acceptance |  | **26** | **92.9** |  |
| Accepting functional, lifestyle and social changes following the tumour and its treatment. | *Accepting new health behaviours* | 6 | 21.4 | Pa32: I mean I’m on about 400mg. It’s something called Carbamazepine. I think I’ll always have to have it for the rest of my life. (aged 46, F, grade 3 oligodendroglioma, 14 years since diagnosis) |
|  | *Accepting social difficulties§* | 0 | 0 |  |
|  | *Accepting support** | 3 | 10.7 | Pa17: I had to accept practical help, emotional help. And I struggled with that for a long time. And I kept wanting to be… I wasn’t a very good patient at the beginning, you know, because that losing of independence is massive. (aged 51, F, grade 3 oligodendroglioma, 8 years since diagnosis) |
|  | *Accepting the tumour and its consequences* | 26 | 92.9 | Pa9: There’s nothing that you can do. It is just that it is what it is, and you’ve just got to make the best of it. (aged 22, M, grade 2 astrocytoma, 1 year since diagnosis)  Pa26: If you’ve got a brain tumour you know your life expectancy is going to be shot a little bit, so don’t focus on that. (aged 37, F, grade 2 oligodendroglioma, 15 years since diagnosis) |
| 1. Activity-based coping |  | **19** | **67.9** |  |
| Use or uptake of hobbies or activities to manage one’s wellbeing. | *Pursuing an existing hobby/activity* | 19 | 67.9 | Pa18: Yes, so music, cooking. I love cooking. Been decorating the house. Some people say I have a good eye. I think I’ve got a very good eye. (aged 55, F, grade 3 oligodendroglioma, 1 year since diagnosis)  Pa33: I mean I love my reading. I read a lot and I’ve got no problem with that. I watch quite a lot of films. (aged 45, M, grade 2 oligodendroglioma, 9 years since diagnosis) |
|  | *Taking up a new hobby/activity* | 8 | 28.6 | Pa35: I had bought myself lots of pieces and started building myself an aeroplane. I’m finally finishing it right now which is exciting. (aged 49, M, grade 2 astrocytoma, 18 years since diagnosis) |
| 1. Adopting a healthy lifestyle |  | **26** | **92.9** |  |
| Adopting generic health behaviours to boost one’s general physical and/or emotional wellbeing. | *Adopting a healthy diet* | 4 | 14.3 | Pa13: keep the iron up, a lot of kale, a lot of spinach… so I researched diet and I decided to…take control. (aged 52, M, grade 3 oligodendroglioma, 13 years since diagnosis) |
|  | *Avoiding negative health behaviours§* | 0 | 0 |  |
|  | *Being physically active in everyday life* | 19 | 67.9 | Pa9: Yeah. So, I mean, I used to walk and still try to walk every, like, all the time, every day. (aged 22, M, grade 2 astrocytoma, 1 year since diagnosis)  Pa36: My husband and I play tennis. We play squash. What else do we do? We swim. (aged 42, F, grade 2 astrocytoma, 8 years since diagnosis) |
|  | *Drinking more water* | 1 | 3.6 | Pa34: So I make sure I drink plenty now. All of the time I’m drinking and it seems to help. (aged 66, F, grade 2 oligodendroglioma, 11 years since diagnosis) |
|  | *Ensuring personal hygiene§* | 0 | 0 |  |
|  | *Exercising* | 16 | 57.1 | Pa11: I’ve always been relatively fit and I used my exercise as my stress buster and whatever. (aged 57, M, grade 2 oligodendroglioma, 14 years since diagnosis)  Pa17: I do yoga. I actually do more since because I now do it almost daily from the TV, whereas when I went to a class it was once a week. (aged 51, F, grade 3 oligodendroglioma, 8 years since diagnosis) |
|  | *Meditating* | 4 | 14.3 | Pa36: Mindfulness is a huge thing which my therapist introduced me to about five years ago. I’ve done lots of mindfulness in various guises. (aged 42, F, grade 2 astrocytoma, 8 years since diagnosis) |
|  | *Reducing negative health behaviours§* | 0 | 0 |  |
|  | *Sleeping well* | 1 | 3.6 | Pa18: Eat well, sleep well, take some gentle exercise, walk the dog, get on the bike. (aged 55, F, grade 3 oligodendroglioma, 1 year since diagnosis) |
|  | *Taking medication* | 18 | 64.3 | Pa26: Just the epilepsy pills. I take four in the morning and four in the evening. (aged 37, F, grade 2 oligodendroglioma, 15 years since diagnosis)  Pa37: I take two tablets of one drug twice a day and another two tablets of another drug twice a day. (aged 54, M, grade 2 astrocytoma, 3 years since diagnosis) |
|  | *Taking vitamins and minerals* | 1 | 3.6 | Pa17: I’ve got to take all sorts of supplements. (aged 51, F, grade 3 oligodendroglioma, 8 years since diagnosis) |
| 1. Behavioural avoidance |  | **8** | **28.6** |  |
| Behavioural strategies which minimise one’s contact with threats to one’s physical and/or emotional wellbeing. | *Avoiding activities that may cause harm* | 4 | 14.3 | Pa31: Don’t read the internet. That is a nightmare. If you read that, you’re on deaths door and it’s not like that for all of them. (aged 53, M, grade 2 oligodendroglioma, 14 years since diagnosis) |
|  | *Avoiding contact with others for possible infection§* | 0 | 0 |  |
|  | *Avoiding situations that may cause harm* | 2 | 7.1 | Pa5: It’s just this sort of brain flooding. If we have a lot of people over for a meal or something like that. I do sometimes take myself off because it gets a bit, there’s too much going on, the brain has taken too much. (aged 56, M, grade 2 oligodendroglioma, 2 years since diagnosis) |
|  | *Avoiding uncomfortable social encounters* | 5 | 17.9 | Pa3: I suppose, because I’ve been so lucky with having such a minimal impact, and I’ve got a very low-grade diagnosis, and I’ve had a lot of healthy years, there is also part of me that is quite afraid of being in an environment where you meet other people who are suffering much more or have a much worse diagnosis. (aged 45, M, grade 2 oligodendroglioma, 18 years since diagnosis) |
| 1. Cognitive avoidance |  | **20** | **71.4** |  |
| Strategies involving the avoidance of thoughts concerning the negative consequences of the tumour and its treatment. | *Avoiding finding out too much* | 9 | 32.1 | Pa38: No. I’m one of these people that probably prefers not to know what's coming, to be honest with you. (aged 55, F, grade 2 astrocytoma, 1 year since diagnosis) |
|  | *Avoiding thoughts about the tumour and its consequences* | 18 | 64.3 | Pa3: I am happy, normally, to talk about it quite openly, if someone asks, but it is, there is an emotional effort in not letting it take over, you know, your life and how you think about things. (aged 45, M, grade 2 oligodendroglioma, 18 years since diagnosis)  Pa18: I forget. I forget because what’s the point in walking around with a sticker on your forehead saying I’ve got a brain tumour? (aged 55, F, grade 3 oligodendroglioma, 1 year since diagnosis) |
|  | *Dealing with (in)fertility at the right time§* | 0 | 0 |  |
|  | *Distracting oneself by keeping busy* | 2 | 7.1 | Pa10: I think I’ve often used work as a distraction. (aged 37, F, grade 2 oligodendroglioma, 14 years since diagnosis) |
| 1. Conserving emotional energy |  | **13** | **46.4** |  |
| Strategies which enable one to conserve emotional energy in order to better self-manage one’s condition. | *Caring less about what others think§* | 0 | 0 |  |
|  | *Having time to yourself* | 3 | 10.7 | Pa18: I dedicate more time to myself. I give myself time to get ready and whatever. (aged 55, F, grade 3 oligodendroglioma, 1 year since diagnosis) |
|  | *Letting emotions out* | 4 | 14.3 | Pa26: There was lots of swearing and literally I just spoke to my dad and partner, leave me alone for about three hours. I need to vent. (aged 37, F, grade 2 oligodendroglioma, 15 years since diagnosis) |
|  | *Minimising stress* | 5 | 17.9 | Pa36: A very big piece I think for me and I’m sure other brain tumour sufferers is stress and just trying to manage your stress…don’t put too much stress or pressure on yourself. (aged 42, F, grade 2 astrocytoma, 8 years since diagnosis) |
|  | *Switching off* | 1 | 3.6 | Pa28: Other things are piled on you, you really do think, well yeah, I’ve had enough. It does sometimes get to that point, yes. I’ve had enough of all this yes and the best thing at that time is to blast out some music or go for a walk down the seafront. (aged 66, M, grade 2 astrocytoma, 7 years since diagnosis) |
|  | *Using sleep* | 5 | 17.9 | Pa35: I’m trying to spend more time sleeping, resting, being less tired. (aged 49, M, grade 2 astrocytoma, 18 years since diagnosis) |
| 1. Conserving physical energy |  | **18** | **64.3** |  |
| Strategies which enable one to conserve physical energy in order to better self-manage one’s condition. | *Reducing activities* | 3 | 10.7 | Pa40: I just try and agree to do a little bit less than I would have done before to try and stop myself getting worn out (aged 31, F, grade 2 astrocytoma, 1 year since diagnosis) |
|  | *Reducing workload* | 14 | 50 | Pa3: “Well, actually, I’d quite like to go part-time, just to…” you know, just to give myself a bit more breathing space in my work week, kind of thing. (aged 45, M, grade 2 oligodendroglioma, 18 years since diagnosis)  Pa30: Starting with half days originally, five half days. Then it would be two full days and gradually build it up until you felt like you could do your seven. If at any time I felt like I couldn’t do it, I could just say, “I need to stop.” (aged 61, M, grade 3 oligodendroglioma, 12 years since diagnosis) |
|  | *Taking a break* | 13 | 46.4 | Pa26: There’s a pond round the corner, I say just round corner but there were times it took me nearly two hours to get there and back, I didn’t complete it every time, I’d have two days rest and then go again and go a bit further each time. (aged 37, F, grade 2 oligodendroglioma, 15 years since diagnosis)  Pa36: If I have a quick nap for half an hour, that usually rejuvenates me. (aged 42, F, grade 2 astrocytoma, 8 years since diagnosis) |
| 1. Creating a healthy environment |  | **28** | **100** |  |
| Attempts to create an environment which enables effective self-management. | *Acquiring knowledge about the tumour, treatment and late effects and available support* | 19 | 67.9 | Pa5: The brain tumour charity website’s really good for the particular… You know if you put in oligo grade 2, diffuse, you get a lot of information about that actually. (aged 56, M, grade 2 oligodendroglioma, 2 years since diagnosis)  Pa10: I just looked for health anxiety therapist or something like that in Google, like, local to [City] and I found her that way. (aged 37, F, grade 2 oligodendroglioma, 14 years since diagnosis) |
|  | *Attending follow-up and screening appointments* | 21 | 75 | Pa13: We have routine… yeah, there are scans built-in, you know, sort of, every three months. And every time we see the oncologist, we usually book in the next scan at the same time. (aged 52, M, grade 3 oligodendroglioma, 13 years since diagnosis)  Pa22: I was going for regular scans. (aged 43, F, grade 2 astrocytoma, 16 years since diagnosis) |
|  | *Collecting materials to aid self-management* | 9 | 32.1 | Pa18: PIP I was given a Macmillan pack at the hospital. I brought that home, read through it. (aged 55, F, grade 3 oligodendroglioma, 1 year since diagnosis) |
|  | *Ensuring reliability of health information on the internet* | 6 | 21.4 | Pa5: You get to know actually which are the websites and which are the people you want to take information from and those which you think, no, you don’t really know what you’re talking about. (aged 56, M, grade 2 oligodendroglioma, 2 years since diagnosis) |
|  | *Learning self-management skills* | 4 | 14.3 | Pa30: Because it affects my left hand side, the tumour, I was always right handed anyhow, I’ve started using my left hand as much as I can to do things. I don’t know whether it helps or not but it helps me. (aged 61, M, grade 3 oligodendroglioma, 12 years since diagnosis) |
|  | *Obtaining resources to aid self-management* | 16 | 57.1 | Pa15: I've got my disabled person's rail pass, I've got the local bus pass. (aged 55, M, grade 2 astrocytoma, 7 years since diagnosis)  Pa36: For relaxation, when I rest I have the Calm app. I’ll put that music or meditation on and listen to that. (aged 42, F, grade 2 astrocytoma, 8 years since diagnosis) |
|  | *Relationship-building with health practitioner* | 3 | 10.7 | Pa18: I found right from the start is that develop good relationships with these people. They’re working with you. They have their expertise. My expertise is elsewhere. But develop really good relationships. (aged 55, F, grade 3 oligodendroglioma, 1 year since diagnosis) |
|  | *Using external aids to overcome cognitive difficulties* | 16 | 57.1 | Pa29: I’ve got a big calendar that I write everything on in the kitchen. I just keep on top of things that way. (aged 51, F, grade 3 oligodendroglioma, 9 years since diagnosis)  Pa38: I thought, “Oh, God, I forgot to take that medicine.” So, my tablet. So, we've actually set the Alexa down the stairs in the living room. So, she's got an alarm at both times, just in case. (aged 55, F, grade 2 astrocytoma, 1 year since diagnosis) |
|  | *Utilising skills for independent living* | 3 | 10.7 | Pa3: I’ll tap my back pocket, and I should be able to hear my pills rattling. And if I can’t feel the pill box in there, then something is wrong, and I’ve got to go back home and get my pills… sort of like a habit. (aged 45, M, grade 2 oligodendroglioma, 18 years since diagnosis) |
|  | *Valuing and respecting relationship with care team* | 3 | 10.7 | Pa28: [Consultant]’s team, the nurses, you can ring them anytime. I’ve got their times and their numbers pinned up in the kitchen there, so, that is like a little support team in itself so that’s useful. (aged 66, M, grade 2 astrocytoma, 7 years since diagnosis) |
| 1. Goal and action setting |  | **25** | **89.3** |  |
| Use of planning or goal-setting self-management strategies. | *Coping planning* | 3 | 10.7 | Pa36: I think it’s just the approach of taking one step at a time. (aged 42, F, grade 2 astrocytoma, 8 years since diagnosis) |
|  | *Planning daily activities* | 8 | 28.6 | Pa30: I’ve just been pottering round as it was trying to achieve something every day, no matter what it is or how small. (aged 61, M, grade 3 oligodendroglioma, 12 years since diagnosis) |
|  | *Priority-based planning* | 7 | 25 | Pa18: Well I’ve got things to look forward to and the first thing is rebuilding myself inside out. (aged 55, F, grade 3 oligodendroglioma, 1 year since diagnosis) |
|  | *Setting future goals* | 18 | 64.3 | Pa13: The swimming is… the aim is to try and get good enough to do a sponsored swim. (aged 52, M, grade 3 oligodendroglioma, 13 years since diagnosis)  Pa31: If you have three, well three and a bit, medical licences, you get your ten-year licence back. It’s something to look forward to in my eyes. Well, it was for me. (aged 53, M, grade 2 oligodendroglioma, 14 years since diagnosis) |
|  | *Setting up facilitating conditions* | 18 | 64.3 | Pa16: I’ve got lists coming out of my ears. I’ve got Google Keep lists, I’ve got a daybook, I’ve got a little notebook. (aged 69, M, grade 3 oligodendroglioma, 2 years since diagnosis)  Pa33: I said to my wife, I said, “I want another box so I can keep two weeks’ worth of medication going just to give me a bit more notice of when I’m getting low.” (aged 45, M, grade 2 oligodendroglioma, 9 years since diagnosis) |
| 1. Managing others |  | **22** | **78.6** |  |
| Active attempts to effectively manage one’s social relationships following treatment. | *Avoidance of negative relationships§* | 0 | 0 |  |
|  | *Being assertive in social encounters* | 1 | 3.6 | Pa31: Some things, I would never say things once upon a time, but now I will if I need to. (aged 53, M, grade 2 oligodendroglioma, 14 years since diagnosis) |
|  | *Being open with others about the tumour and its consequences* | 18 | 64.3 | Pa15: I'm really open with everybody about it, everybody knows it, everybody knows I suffer from epilepsy. It's not a secret. (aged 55, M, grade 2 astrocytoma, 7 years since diagnosis)  Pa29: With friends and that kind of thing, there are certain ones that I know that I could talk to about it and that would be fine. (aged 51, F, grade 3 oligodendroglioma, 9 years since diagnosis) |
|  | *Keeping others happy* | 5 | 17.9 | Pa25: There was other things I used to be a part of, but I get a little bit sort of wary about making life difficult for them. You know, these people don’t know anything or don’t want to deal with somebody who’s got cancer. (aged 45, M, grade 2 oligodendroglioma, 11 years since diagnosis) |
|  | *Protecting others from harm* | 18 | 64.3 | Pa11: I suppose when she realised, obviously, when she was a bit younger, we didn’t say it was a brain tumour or whatever, just said daddy’s got a wee, funny spells and whatever, and that was good enough for her. (aged 57, M, grade 2 oligodendroglioma, 14 years since diagnosis)  Pa17: I still feel the need to protect others by being positive even when I don’t feel it. (aged 51, F, grade 3 oligodendroglioma, 8 years since diagnosis) |
| 1. Meaning-making |  | **27** | **96.4** |  |
| Interpreting the tumour and its consequences in the broader context of life as a whole. | *Appreciating health more* | 4 | 14.3 | Pa30: That is what well for me is. As long as I can keep my mobility and things like that I’ll be quite happy. (aged 61, M, grade 3 oligodendroglioma, 12 years since diagnosis) |
|  | *Appreciating life more* | 10 | 35.7 | Pa17: Your appreciation suddenly becomes, you know… you stop taking things for granted. You don’t waste time as you can probably tell, I don’t. And I put live again. Don’t waste time. (aged 51, F, grade 3 oligodendroglioma, 8 years since diagnosis) |
|  | *Appreciating support* | 15 | 53.6 | Pa9: I don’t know what I’d do if it weren’t for my mum. She always rings up the hospital, and I probably wouldn’t even of had the operation. (aged 22, M, grade 2 astrocytoma, 1 year since diagnosis)  Pa15: I'm lucky to have such supportive family and friends, and such a brilliant medical team looking after me. I'm really lucky. (aged 55, M, grade 2 astrocytoma, 7 years since diagnosis) |
|  | *Appreciating the importance of family* | 5 | 17.9 | Pa33: My kids, they are my prime… any time I have when I’m feeling good, I want to spend it with my kids. (aged 45, M, grade 2 oligodendroglioma, 9 years since diagnosis) |
|  | *Appreciating the severity of one’s illness history* | 7 | 25 | Pa13: You know, it’s thirteen years, and the amount of treatments… all the chemo, all the radiation… and, you know, having my brain chopped open three times… Yeah, I’m still quite impressed with my physical robustness. (aged 52, M, grade 3 oligodendroglioma, 13 years since diagnosis) |
|  | *Becoming more altruistic§* | 0 | 0 |  |
|  | *Changing one’s image* | 1 | 3.6 | Pa34: I was more empathetic with patients who had other conditions as well as kidney stones because it made me think, “Well if I had this and kidney stones which would be the more dominant thing to think about?”... I think I was probably a better nurse because I’d had this experience myself. (aged 66, F, grade 2 oligodendroglioma, 11 years since diagnosis) |
|  | *Finding meaning in work* | 4 | 14.3 | Pa15: I missed work when I was off. They're long, lonely days especially when the weather's crap and you can't do much. So, getting back to work with colleagues that I value and trust and enjoy working with, was a real kind of boost for me. (aged 55, M, grade 2 astrocytoma, 7 years since diagnosis) |
|  | *Giving back* | 10 | 35.7 | Pa11: I set up, with the help of a few friends, we did a couple of running events, and we raised about £25,000 for charity. (aged 57, M, grade 2 oligodendroglioma, 14 years since diagnosis)  Pa16: I like to help people, if I can share their experience and bring my positive attitude into their life a little bit, then that’s good. (aged 69, M, grade 3 oligodendroglioma, 2 years since diagnosis) |
|  | *Taking every day as it comes* | 12 | 42.9 | Pa29: I think we’re quite practical and so things like that, we deal with it as and when it comes. (aged 51, F, grade 3 oligodendroglioma, 9 years since diagnosis)  Pa37: I just try to take each day as it comes and that’s the way I cope with it. (aged 54, M, grade 2 astrocytoma, 3 years since diagnosis) |
|  | *Wanting to give something back* | 12 | 42.9 | Pa18: I said, “When I come back, I’d really like to talk to people in the company who are either supporting, caring for somebody who’s unwell or somebody who is going through a really crappy time through either health or whatever.” (aged 55, F, grade 3 oligodendroglioma, 1 year since diagnosis)  Pa26: I’d love to sit and help someone or just listen to their problems rather than it being mine. (aged 37, F, grade 2 oligodendroglioma, 15 years since diagnosis) |
| 1. Positive appraisal |  | **26** | **92.9** |  |
| Focusing on positive aspects of one’s immediate situation. | *Benefit finding* | 13 | 46.4 | Pa5: In some ways the tumour’s been a good thing. It’s given me an excuse to rest. Before I probably would have got a bit het up and frustrated I wasn’t doing all the stuff on this list. (aged 56, M, grade 2 oligodendroglioma, 2 years since diagnosis)  Pa17: None of my good friends abandoned me. And I’ve actually gained a lot more friendships since, you know. (aged 51, F, grade 3 oligodendroglioma, 8 years since diagnosis) |
|  | *Downward comparison* | 18 | 64.3 | Pa10: Some patients have horrible side effects from their surgery or from their chemo or whatever whereas I’ve never had that, luckily. (aged 37, F, grade 2 oligodendroglioma, 14 years since diagnosis)  Pa19: I’ve got to make decisions and I imagine because most people are dead within ten years with what I’ve got, 80% of people are dead within ten years. I’m now coming to my eleventh year. (aged 55, M, grade 3 oligodendroglioma, 5 years since diagnosis) |
|  | *Reinterpreting negative consequences* | 24 | 85.7 | Pa13: When I came out, I thought, “You know, a wheelchair is not too bad. Wheelchair guys are pretty buff. And wheelchair rugby looks like a good sport.” So I had these visions of becoming a Paralympian. (aged 52, M, grade 3 oligodendroglioma, 13 years since diagnosis)  Pa33: I retired medically, got all the stuff done with occ health and all the rest of it. It’s the best thing I’ve ever done, retiring. It’s the best thing I’ve ever done because at least now I can have some life with my kids and my wife. (aged 45, M, grade 2 oligodendroglioma, 9 years since diagnosis) |
| 1. Proactive problem solving |  | **18** | **64.3** |  |
| Active attempts to solve problems in-the-moment arising from the consequences of the tumour and its treatment. | *Acting to prevent further complications* | 7 | 25 | Pa14: I was prompt to complete the six-page form to reapply for the reinstatement of my licence and I submitted that in good time along with supporting documentation. (aged 66, M, grade 2 oligodendroglioma, 4 years since diagnosis) |
|  | *Adaptive approaches to ongoing physical consequences of the tumour and its treatment* | 17 | 60.7 | Pa29: I can walk to the supermarket at the end of our street. I could live without driving the car. (aged 51, F, grade 3 oligodendroglioma, 9 years since diagnosis)  Pa33: Even just coming up and down the stairs, I try and bring everything down that I need for the day so I don’t have to go back up and down the stairs. (aged 45, M, grade 2 oligodendroglioma, 9 years since diagnosis) |
| 1. Reasoned decision-making |  | **23** | **82.1** |  |
| Objective decision-making strategies relating to survivor self-management. | *Considering benefits of positive health behaviours* | 9 | 32.1 | Pa22: I was going to the exercise class at the leisure centre. That was just once a week. I did feel that did me good just to get out the house. (aged 43, F, grade 2 astrocytoma, 16 years since diagnosis) |
|  | *Considering pros and cons of self-management* | 9 | 32.1 | Pa19: I’m writing and I think that has really helped my brain. I think if I was sitting down watching TV, oh I think I’d be all over the place. I think this focuses my brain. (aged 55, M, grade 3 oligodendroglioma, 5 years since diagnosis) |
|  | *Evaluating effectiveness of self-management* | 10 | 35.7 | Pa11: I think maybe that’s the whole process of basically speaking through things over the years. And now, I just feel completely comfortable with the whole situation. So, no, it’s good. (aged 57, M, grade 2 oligodendroglioma, 14 years since diagnosis)  Pa30: I’ve been doing things on my own, word searches, brain tests…sometimes helps. (aged 61, M, grade 3 oligodendroglioma, 12 years since diagnosis) |
|  | *Thinking objectively about negative health behaviours* | 1 | 3.6 | Pa35: I definitely work too hard and I’ve got to try and reduce that by my partner’s right. (aged 49, M, grade 2 astrocytoma, 18 years since diagnosis) |
|  | *Thinking objectively about negative thoughts and emotions* | 9 | 32.1 | Pa26: If I get too upset and I over think things and I get too down that can then trigger my seizures and then that means I lose my license. (aged 37, F, grade 2 oligodendroglioma, 15 years since diagnosis) |
| 1. Seeking normality |  | **23** | **82.1** |  |
| Active attempts to return to normal living following treatment. | *Balancing life with health needs§* | 0 | 0 |  |
|  | *Carrying out tasks to the best of one’s ability* | 5 | 17.9 | Pa19: I try to do as much as I can, but I have to be realistic about my energy, having so much energy. (aged 55, M, grade 3 oligodendroglioma, 5 years since diagnosis) |
|  | *Choosing when and to whom to disclose illness history* | 2 | 7.1 | Pa28: I think the best friends don’t really ask for much about what’s going on. I think they don’t really say, oh, how’s your tumour today and I think probably our best friends have never really asked about it which I think that’s quite a good thing. (aged 66, M, grade 2 astrocytoma, 7 years since diagnosis) |
|  | *Focusing on doing normal activities* | 8 | 28.6 | Pa18: I’ll cook dinner because I choose to. I’ll make sure the washing has been done. (aged 55, F, grade 3 oligodendroglioma, 1 year since diagnosis) |
|  | *Focusing on getting back to work* | 13 | 46.4 | Pa11: I’m one of these stubborn people that, the one constant, because I worked since I was 17 and the one constant is work for me. So, I basically got back to work as quickly as I could. (aged 57, M, grade 2 oligodendroglioma, 14 years since diagnosis)  Pa32: I wanted to be at work because I wanted my life to be normal. I needed that normality to get through stuff as well. (aged 46, F, grade 3 oligodendroglioma, 14 years since diagnosis) |
|  | *Gaining independence* | 4 | 14.3 | Pa34: I think I’m fiercely independent, more independent now because of the diagnosis. I like to be able to do things myself. (aged 66, F, grade 2 oligodendroglioma, 11 years since diagnosis) |
|  | *Maintaining independence* | 3 | 10.7 | Pa29: I’m still competent enough that I can read the website and the letters that they sent me and understand what I need to do. (aged 51, F, grade 3 oligodendroglioma, 9 years since diagnosis) |
|  | *Regaining strength* | 6 | 21.4 | Pa15: I've also continued my physiotherapy for the best part of a year, for the remainder of 2014, which effectively got my fitness back and my strength back. (aged 55, M, grade 2 astrocytoma, 7 years since diagnosis) |
|  | *Returning to normal* | 10 | 35.7 | Pa3: Before you know it, you’re sitting in front of the TV and watching box sets again, like you always were. (aged 45, M, grade 2 oligodendroglioma, 18 years since diagnosis)  Pa18: I don’t think you need to change everything. It’s about keeping a sense of normality, yes, mum’s got this but listen, hey, see, I’m doing really well. (aged 55, F, grade 3 oligodendroglioma, 1 year since diagnosis) |
|  | *Testing oneself* | 1 | 3.6 | Pa26: The lady who was doing the physio, she left and it wasn’t really continued at the hospital or it was a hospital fairly near, so I started making my own with a ball, walking little bits and seeing if I can get up the stairs. (aged 37, F, grade 2 oligodendroglioma, 15 years since diagnosis) |
|  | *Trying to fit in§* | 0 | 0 |  |
| 1. Self-monitoring |  | **27** | **96.4** |  |
| Active self-monitoring of one’s health, wellbeing and ongoing care. | *Knowing your body* | 7 | 25 | Pa19: I’m in charge of my own body. I’m not going to ignore what you say but I know my body. I know my body. (aged 55, M, grade 3 oligodendroglioma, 5 years since diagnosis) |
|  | *Monitoring emotions* | 23 | 82.1 | Pa18: I think it’s about managing your emotions, being able to talk myself down if I’m panicking. (aged 55, F, grade 3 oligodendroglioma, 1 year since diagnosis)  Pa35: I suppose there’s a small amount of anxiety but it’s definitely manageable. It’s not something I’m going to lose sleep about. (aged 49, M, grade 2 astrocytoma, 18 years since diagnosis) |
|  | *Monitoring for symptoms of the tumour and late-effects* | 15 | 53.6 | Pa5: The brain flooding thing, if we have a lot of people over as I said earlier or if I was in an open plan office that was really problematic. I really felt myself just going downhill quite quickly and feeling like I couldn’t speak properly. (aged 56, M, grade 2 oligodendroglioma, 2 years since diagnosis)  Pa14: The other day I was sat just quietly reading something and I was aware that [pause] I was having tinnitus and I thought: “Oh dear, I hope this isn’t something sinister coming back.” (aged 66, M, grade 2 oligodendroglioma, 4 years since diagnosis) |
|  | *Monitoring general health* | 4 | 14.3 | Pa36: So I think when you have a tumour, you’re always like, “Is this a bit sore today? Am I not quite right or a little bit tired?” (aged 42, F, grade 2 astrocytoma, 8 years since diagnosis) |
|  | *Monitoring health behaviours* | 1 | 3.6 | Pa17: I need something creative, I need something for exercise, I need somethings for my mind, you know, you need to think of everything and make sure you’ve got things in all your boxes. (aged 51, F, grade 3 oligodendroglioma, 8 years since diagnosis) |
|  | *Monitoring relationship with health professionals* | 1 | 3.6 | Pa28: If I had a closer relationship… yes, that’s the right word, closer links with the GP surgery I’d probably feel a bit better, but I can’t say there’s any specific reason why I would need that. (aged 66, M, grade 2 astrocytoma, 7 years since diagnosis) |
|  | *Recognising one’s own limits* | 21 | 75 | Pa13: I wouldn’t get on a bike, you know. My balance isn’t… you know, there are certain things that would be dumb. (aged 52, M, grade 3 oligodendroglioma, 13 years since diagnosis)  Pa16: I wouldn’t take on anything I wouldn’t… I couldn’t manage, if there was an opportunity, I would investigate what was involved and see whether I felt I could do it well enough. (aged 69, M, grade 3 oligodendroglioma, 2 years since diagnosis) |
| 1. Self-motivating |  | **25** | **89.3** |  |
| Strategies which help to motivate oneself to effectively self-manage. | *Being healthy for sake of one’s family* | 2 | 7.1 | Pa15: So, my biggest challenge is to make sure that I keep giving her support and try to look after myself so I don't put her in a position whereby she's got to pick up the pieces again. (aged 55, M, grade 2 astrocytoma, 7 years since diagnosis) |
|  | *Challenging yourself* | 2 | 7.1 | Pa11: I set myself my own personal challenge which was doing one 10k a week for a year. And that doesn’t sound like a lot, because I used to run 10ks every day. And I thought that will be easy. But I didn’t take into account that I started it when I was just starting my chemotherapy. So, it was hard. (aged 57, M, grade 2 oligodendroglioma, 14 years since diagnosis) |
|  | *Developing confidence and self-efficacy* | 1 | 3.6 | Pa31: I was quite shy and quite introverted at the time, before, but now it kind of gave me a bit more confidence. (aged 53, M, grade 2 oligodendroglioma, 14 years since diagnosis) |
|  | *Drawing on spiritual resources* | 1 | 3.6 | Pa14: There have been some kind of spiritual and interpersonal aspects to the experience that have been an important boost and support to me. (aged 66, M, grade 2 oligodendroglioma, 4 years since diagnosis) |
|  | *Drawing strength from past experiences* | 2 | 7.1 | Pa34: I’m more in control now than I think I was earlier and it’s because of my experience of the tumour I think. (aged 66, F, grade 27 oligodendroglioma, 11 years since diagnosis) |
|  | *Employing a determined attitude* | 14 | 50 | Pa13: I managed to get myself to bed every time without using the stair-lift. It was a personal sense of achievement…I’m a stubborn bugger. (aged 52, M, grade 3 oligodendroglioma, 13 years since diagnosis)  Pa32: You just have to think, “No, I’m not going to die. I’m going to get through this. It’s not easy but at the same time I’m not going to let it beat me.” (aged 46, F, grade 3 oligodendroglioma, 14 years since diagnosis) |
|  | *Encouraging oneself* | 6 | 21.4 | Pa17: You know, you really find your zest for life. As long as you don’t let it beat you. I know some people sink into depression and they just can’t rise above that. And I’m just glad I’m not in that position. (aged 51, F, grade 3 oligodendroglioma, 8 years since diagnosis) |
|  | *Focusing on milestones of survivorship* | 3 | 10.7 | Pa14: I feel very satisfied with it. I’m waiting to hear further… I think I would be due for a scan, they were scanning me…every four months and now I think since then I’ve had nearly a year of being clear since surgery. (aged 66, M, grade 2 oligodendroglioma, 4 years since diagnosis) |
|  | *Interacting with others* | 1 | 3.6 | Pa19: It was quiet. I thought well let’s kick off with starting something about how it started. I spoke for about 30 seconds or something. Then these two other people said that they’d got it. I thought brilliant that’s three of us, but some people didn’t speak at all. (aged 55, M, grade 3 oligodendroglioma, 5 years since diagnosis) |
|  | *Maintaining a positive outlook* | 20 | 71.4 | Pa18: If I have a good night and the power of positive thinking and a general air of positivity and hopefulness, I’ll start the next day. (aged 55, F, grade 3 oligodendroglioma, 1 year since diagnosis)  Pa33: Emotionally, I try and look at things just as positively as I can. I have two small kids. I’ve got my wife. I can’t just go on moping about stuff so I just try and stay positive emotionally. I just try and stay positive. (aged 45, M, grade 2 oligodendroglioma, 9 years since diagnosis) |
|  | *Not dwelling on the past* | 2 | 7.1 | Pa26: I don’t really want to look back and see how tough it’s been. I like to look forward. (aged 37, F, grade 2 oligodendroglioma, 15 years since diagnosis) |
|  | *Persevering with healthy behaviours* | 3 | 10.7 | Pa30: At the moment I don’t know whether it’s my walking that’s got worse or my energy level, I don’t know. I’m just not getting there at the moment but I keep trying. (aged 61, M, grade 3 oligodendroglioma, 12 years since diagnosis) |
|  | *Recognising the need for motivation and discipline* | 1 | 3.6 | Pa31: I should really get on the bike but it’s finding the time and the inclination to do that. (aged 53, M, grade 2 oligodendroglioma, 14 years since diagnosis) |
|  | *Rewarding oneself§* | 0 | 0 |  |
|  | *Taking responsibility for own health* | 11 | 39.3 | Pa9: In the end, only you can make the end decision. And that’s a responsibility that I’m going to have to bear. (aged 22, M, grade 2 astrocytoma, 1 year since diagnosis)  Pa17: Because of my previous job as a social worker, I was quite proactive in my own, you know, finding answers and not accepting, you know, nothing. (aged 51, F, grade 3 oligodendroglioma, 8 years since diagnosis) |
|  | *Treating illness as a project§* | 0 | 0 |  |
|  | *Wanting to look good§* | 0 | 0 |  |
|  | *Wanting to stay in good health* | 1 | 3.6 | Pa14: Given the age at which I am, it’s made me be more reflective, shall we say, or contemplating what I need to care for myself, to keep optimum health and activity. (aged 66, M, grade 2 oligodendroglioma, 4 years since diagnosis) |
| 1. Self-sustaining |  | **12** | **42.9** |  |
| Strategies which enable one to consistently implement self-management strategies in one’s daily life. | *Customizing dietary practices* | 2 | 7.1 | Pa30: I only spoke to a dietician about foods to eat for fatigue. My go to food was, to try and keep my energy levels up was peanut butter when I couldn’t eat anything else. (aged 61, M, grade 3 oligodendroglioma, 12 years since diagnosis) |
|  | *Following health practitioner’s advice* | 4 | 14.3 | Pa30: I couldn’t steady my hip so she gave me some exercises to do which I carried on doing. (aged 61, M, grade 3 oligodendroglioma, 12 years since diagnosis) |
|  | *Incorporating self-management behaviours into daily routine* | 8 | 28.6 | Pa33: I just make sure I put anything important in my phone calendar and every morning I look at my calendar in my phone. That is a habit now. (aged 45, M, grade 2 oligodendroglioma, 9 years since diagnosis) |
|  | *Keeping busy to avoid negative behaviours* | 2 | 7.1 | Pa11: I suppose work as well, it’s kept me going as well. Because you’re not sort of dwelling on things if you’re keeping yourself busy. And that’s one of the things I’ve tried not to do. (aged 57, M, grade 2 oligodendroglioma, 14 years since diagnosis) |
|  | *Maintaining medical equipment* | 1 | 3.6 | Pa13: I had a port fitted for the chemotherapy. So I have to manage to… you have to get that flushed every month. (aged 52, M, grade 3 oligodendroglioma, 13 years since diagnosis) |
| 1. Using sense of humour |  | **6** | **21.4** |  |
| Use of humour to manage emotions associated with the negative consequences of the tumour and its treatment. | *Finding humour in others' reactions* | 1 | 3.6 | Pa15: I've said a number of times that if I had a pound for everybody who says, “Oh, you have got a brain then?” I say, “Yes, I have. I've seen it, I’ve seen scans, I have got a brain. I might have a bit of a hole in it [laughs]. A bit might be missing but I have got a brain.” It's a joke and that's how we deal with it. (aged 55, M, grade 2 astrocytoma, 7 years since diagnosis) |
|  | *Laughing about the tumour and its consequences* | 5 | 17.9 | Pa29: I keep saying to him, “I don’t want you wiping my bum. Get me on a plane to Dignitas as soon as possible once I realise I’m past it.” I joke about that kind of thing. (aged 51, F, grade 3 oligodendroglioma, 9 years since diagnosis) |
|  | *Using humour to hide insecurities* | 1 | 3.6 | Pa20: I’ve said that about the personality changes. I said, “If I ever get really bad, just stick me in a home, walk away and forget about me.” And he said, “Well, that was the plan anyway, but alright.” (aged 47, F, grade 3 oligodendroglioma, 6 years since diagnosis) |
| 1. Using support |  | **28** | **100** |  |
| Use of appropriate supports to assist in one’s recovery and recuperation following treatment. | *Companionship from pet* | 2 | 7.1 | Pa26: In the field he was a spaniel, but he came with me when I was working. I worked for four children under the age of 10 and he would come with me everywhere. When I had my seizures he would be with me. At my mum and dad’s he would be next to me. (aged 37, F, grade 2 oligodendroglioma, 15 years since diagnosis) |
|  | *Drawing support from similar other* | 15 | 53.6 | Pa13: That’s why Maggie’s is so good. You are dealing with people that, you know… everyone is in the same boat. So, you know, we’re all dealing with it the best we can, so everyone trades tips. (aged 52, M, grade 3 oligodendroglioma, 13 years since diagnosis)  Pa40: I find it most useful in terms of gleaning information that might be helpful or prior to my surgery for instance, hearing people say, “Yes, I’ve had a craniotomy and it was fine”. (aged 31, F, grade 2 astrocytoma, 1 year since diagnosis) |
|  | *Giving advice to similar others** | 7 | 25 | Pa22: Somebody was saying they’d had to surrender their driving licence. I think I suggested, “Well you can get a free bus pass,” and things like that. (aged 43, F, grade 2 astrocytoma, 16 years since diagnosis) |
|  | *Having family and friends to talk to*† (split from having someone to talk to) | 7 | 25 | Pa31: Her parents are still alive. My mother’s still alive. I’ve got brothers, her sister-in-law who lives up in the Borders, then there’s my wife’s sister lives in Australia. The people on this side of the world, they’re always available to talk to if you want them. (aged 53, M, grade 2 oligodendroglioma, 14 years since diagnosis) |
|  | *Having health professionals to talk to*† (split from having someone to talk to) | 16 | 57.1 | Pa5: Obviously there is care going on and they’re there if I need them. The clinical nurse specialists are there and I’ve got a number I can call if there’s any problems. (aged 56, M, grade 2 oligodendroglioma, 2 years since diagnosis)  Pa36: They’re the neuro nurses for the whole department. There are two or three of them and they are brilliant. I email them a lot and they always get back to me or phone me or email me. (aged 42, F, grade 2 astrocytoma, 8 years since diagnosis) |
|  | *Receiving formal support* | 20 | 71.4 | Pa14: The neuro-physiotherapist sent me some exercises to be getting on with. I related to her, so my postural and mobility issues that are limited by symptoms. She sent me a programme. (aged 66, M, grade 2 oligodendroglioma, 4 years since diagnosis)  Pa25: I see a psychiatrist…in the cancer centre. And he’s absolutely fantastic. And he’s always really, really good at being able to give me advice about what I can do if I’ve got a problem. (aged 45, M, grade 2 oligodendroglioma, 11 years since diagnosis) |
|  | *Receiving support from care team* | 19 | 67.9 | Pa10: I could speak to the neuro-oncology nurses and say, “Look, this is what’s happening, what do you think?” And they were like, “Well, we think it’s probably okay. We doubt it’s anything to do with your brain tumour.” So that was reassuring really because nobody else knew what was going on. (aged 37, F, grade 2 oligodendroglioma, 14 years since diagnosis)  Pa18: I think definitely my brain care nurse has been amazing but having one person that can say, “Oh yes, you need to go here,” that’s great. (aged 55, F, grade 3 oligodendroglioma, 1 year since diagnosis) |
|  | *Receiving support from charities and organisations* | 17 | 60.7 | Pa9: I did, like, this webinar, actually, last week and it was about fatigue. So, that was, like, a helpful thing. It was through The Brain Tumour People, I think it’s called. Well, Brain Cancer People. (aged 22, M, grade 2 astrocytoma, 1 year since diagnosis)  Pa26: Macmillan nurses are gold dust. Without them, they gave me a grant when I first had my brain surgery. It wasn’t masses but it was something. (aged 37, F, grade 2 oligodendroglioma, 15 year since diagnosis) |
|  | *Receiving support from educational provider§* | 0 | 0 |  |
|  | *Receiving support from family* | 24 | 85.7 | Pa16: If I’m running out of energy, strength then I can call upon brothers-in-law to come and give me a lift. My two sisters-in-law have been really supportive of my wife, that’s been rather lovely, yeah, been very lovely, yes. (aged 69, M, grade 3 oligodendroglioma, 2 years since diagnosis)  Pa17: When I was poorlier, people cooked for me, they took me out, they drove me. You know, I couldn’t drive, they took me shopping. (aged 51, F, grade 3 oligodendroglioma, 8 years since diagnosis) |
|  | *Receiving support from friends* | 24 | 85.7 | Pa32: I used to go to work in the morning. My friends were just incredible. They set up a little rota to take me there and then I’d do it like that. (aged 46, F, grade 3 oligodendroglioma, 14 years since diagnosis)  Pa37: We have a close relationship with a couple of families if you like or certainly they’re friends of ours shall we say. They’re very, very supportive. Very, very supportive. (aged 54, M, grade 2 astrocytoma, 3 years since diagnosis) |
|  | *Receiving support from partner* | 20 | 71.4 | Pa3: It was kind of fortunate, because my dad and my partner could, kind of, work as, sort of, like, a caring team together, if you like, to sort through it. (aged 45, M, grade 2 oligodendroglioma, 18 years since diagnosis)  Pa33: My wife is brilliant. She supports me fantastically well…I don’t know where I’d be without my wife. (aged 45, M, grade 2 oligodendroglioma, 9 years since diagnosis) |
|  | *Receiving support from the workplace* | 21 | 75 | Pa10: They kept a TA, a teaching assistant in the room with me for the first few days just in case I was unwell or anything like that. (aged 37, F, grade 2 oligodendroglioma, 14 years since diagnosis)  Pa14: Well, I didn’t suffer any penalty financially, my employer continued to pay me my monthly salary as normal. (aged 66, M, grade 2 oligodendroglioma, 4 years since diagnosis) |
|  | *Seeking formal help* | 17 | 60.7 | Pa18: I found out about the EESA because I had a query about child benefit. I thought, “Oh okay,” so it just led me on to seeing if there was anything I was entitled to as somebody with a longstanding illness and that was it. (aged 55, F, grade 3 oligodendroglioma, 1 year since diagnosis)  Pa31: I went in because obviously it was brand new. I didn’t know where I stood financially and all the rest of it. I went in to get some information from there at… the [hospital]. (aged 53, M, grade 2 oligodendroglioma, 14 years since diagnosis) |
|  | *Seeking support from care team* | 13 | 46.4 | Pa14: I rang the clinical nurse specialist to tell them about it. They said thank you for letting us know and we’ll speak with our colleagues, and they came back to me and said: “We would like you to come in,” and I went into clinic and saw them. (aged 66, M, grade 2 oligodendroglioma, 4 years since diagnosis)  Pa20: We’ve also got a hospice in town, and it’s actually not far from here. I’ve been before for acupuncture, and they said that they do counselling and stuff, so I might have to go to them. (aged 47, F, grade 3 oligodendroglioma, 6 years since diagnosis) |
|  | *Seeking support from charities and organisations** | 14 | 50 | Pa17: Brain Trust, if you contact them, they will send you a brain box, which is a shoebox full of useful books, leaflets, contact numbers, stress balls, tea bags. You know, they’re all sponsored by, you know, people donating. (aged 51, F, grade 3 oligodendroglioma, 8 years since diagnosis)  Pa29: I knew there was a Maggie’s Centre there and I think I’d just gone across after one of my scans probably to have a look and had a wander round. (aged 51, F, grade 3 oligodendroglioma, 9 years since diagnosis) |
|  | *Seeking support from family* | 1 | 3.6 | Pa22: I had to ask friends or family and it was that reliance on other people. (aged 43, F, grade 2 astrocytoma, 16 years since diagnosis) |
|  | *Seeking support from friends* | 2 | 7.1 | Pa32: That first night I found out, I did think, “Oh my God. Am I going to die?” It says it all, I probably didn’t like to call mum but I called my friend [name] really early because she gets into work stupid early. I was in tears with her. (aged 46, F, grade 3 oligodendroglioma, 14 years since diagnosis) |
|  | *Seeking support from partner§* | 0 | 0 |  |
|  | *Seeking support from the workplace** | 1 | 3.6 | Pa29: “Get back in touch with [employer]…and see if there’s any way they can retire you on ill health grounds.” So I got in touch with them. (aged 51, F, grade 3 oligodendroglioma, 9 years since diagnosis) |

*M = Male; F = Female*

* New strategy identified in the LGG data

† Original strategy has been sub-divided into new categories

*§* Original strategy not identified in the LGG data
